# The Effectiveness of Tailored Interactive Multimedia Software based on the Trans-Theoretical Model for the Promotion of Physical Activity Behaviours

**DOI:** 10.21315/mjms2022.29.4.13

**Published:** 2022-08-29

**Authors:** Somayeh Jalali, Nasrin Roozbahani, Mohsen Shamsi

**Affiliations:** Department of Health Education, School of Health, Arak University of Medical Sciences, Arak, Iran

**Keywords:** trans-theoretical model, physical activity, multimedia, adult

## Abstract

**Background:**

Although physical activity (PA) is essential, it is difficult to motivate people to take part in PA, especially the ones with high blood pressure (hypertension). One of the most commonly applied theoretical frameworks to change health behaviours is the Trans-Theoretical Model (TTM). The aim of this study was to examine the effectiveness of tailored, interactive multimedia software based on TTM for the promotion of PA behaviours among Iranian adults.

**Methods:**

This interventional study selected 120 healthy individuals aged 30 years old–50 years old from health centres in Delijan, Iran using multi-stage sampling. The participants were in the pre-contemplation and contemplation stages of PA behaviour. For the intervention, an active multimedia training (active life) based on TTM, like six training sessions of approximately 30 min (once a week) plus aerobic exercise videos were designed and provided to the intervention group. Data was collected using a valid and reliable questionnaire before and 6 months after the intervention and analysed using statistical tests.

**Results:**

The mean age of the participants was 38.20 (SD = 7.11) years old. Six months after the intervention, 61.7% of the individuals in the intervention group and 3.3% in the control group progressed to the action stage, which was significant only in the intervention group (*P* < 0.001). There was a significant increase in PA in both groups, yet this increase was significantly higher in the intervention group (36.02 [SD = 31.22] to 146.16 [SD = 90.43]; *P* < 0.001) compared to the control group (33.41 [SD = 28.33] to 54.41 [SD = 44.02; *P* < 0.001).

**Conclusion:**

The results obtained indicated that the implementation of educational intervention using multimedia designed based on TTM could be used as one of the effective strategies to promote PA to prevent hypertension in adults.

## Introduction

Approximately one billion people in the world have hypertension, which is predicted to reach 1.56 billion by 2025 (1). Hypertension is an independent and strong risk factor for cardiovascular disease, renal diseases, stroke and the third leading cause of death in the world, which causes 7.5 million deaths annually (2). According to the Seventh Report of the Joint National Committee on Prevention, Detection, Evaluation and Treatment of High Blood Pressure (JNC 7), adopting a healthy lifestyle is an inevitable part of managing people with hypertension and preventing the disease, one of whose components is regular physical activity (PA) like brisk walking (at least 30 min most days of the week) (3). By changing socioeconomic patterns around the world, sedentary lifestyles have become a global phenomenon (4). Inactivity is on the rise in many countries and is one of the main reasons for the increase in the prevalence of non-communicable diseases and the decline in public health around the world (5–6). It is challenging to motivate people to participate in PA, and many individual and social factors prevent them from engaging in regular PA (7). Analysis of the reasons for poor health-related quality of life in Iran indicated that after socioeconomic status, age, gender and race, the most influential factor is PA. This issue is an indicative of and necessity of administering appropriate policies to improve PA rate among Iranians (8).

The theories and models of health education could be useful in identifying the main factors affecting behaviour and the relationships between them (9). Using these theories and models to design and implement health education programmes could bring about more effective educational interventions in the target groups (10). One of the most prevalent theoretical frameworks used to change health behaviour is Trans-Theoretical Model (TTM) (11). This model shows that individuals go through various stages during behaviour change and that the whole process can take between 6 months and 5 years. These steps are closely related to individual motivation: i) pre-contemplation; ii) contemplation; iii) preparation; iv) action and v) maintenance (12).

Educational multimedia—a combination of speech, music, graphics and text—can be used to increase awareness, develop skills and change behaviour for several reasons like the ability to adjust the training time as wished, cost-effectiveness, the ability to use educational materials frequently and attractiveness of the individuals (13–14). It is necessary to combine health education theories (to determine the content and order of education) and multimedia principles (to determine the correct way to use multimedia) for using this technology effectively. The study was designed and implemented to examine the effectiveness of tailored, interactive multimedia software based on TTM for the promotion of PA behaviours.

## Methods

**Figure 1:**
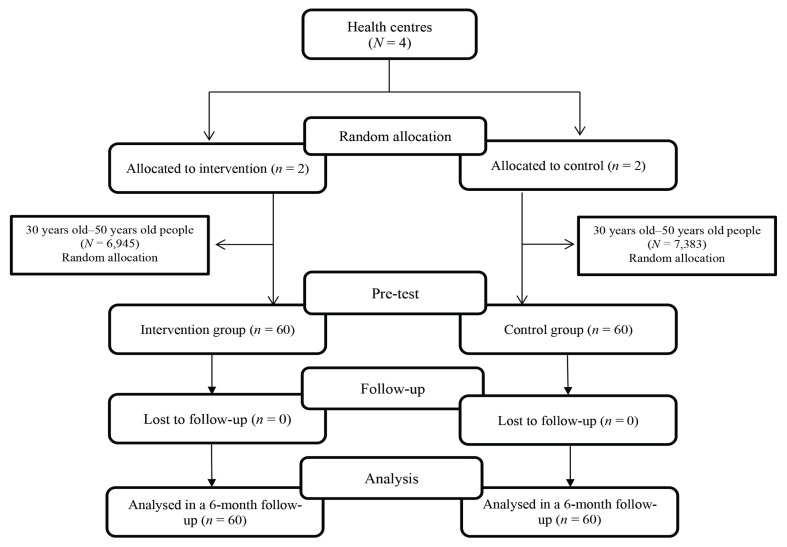
Flow diagram of the participants. From a total of four health centres in Delijan, two centres were randomly assigned (by drawing) to the intervention group and two centres to the control group. According to the recorded information in each center, a list of people aged 30 years old–50 years old was extracted and each person was assigned a number. Later, using a table of random numbers, 60 people were selected for each group (30 people in each centre) and were invited by the health centre.

This clinical trial was done on 120 adults aged 30 years old–50 years old in Delijan in central Iran. At the beginning of the study, the population of Delijan was 40,902 and the population aged 30 years old–50 years old was 14,328. Almost the entire population of the city is covered by health centres and their information has been registered in these centres. Inclusion criteria were adults aged 30 years old–50 years old, being in the stages of changing pre-contemplation and contemplation about PA behaviour, not having maim and mental or physical illnesses like chronic illnesses, and the possibility of using multimedia software. Exclusion criteria were the participants’ lack of interest to continue the study, severe diseases, contraindication to PA or lack of multimedia view. According to the pilot study considering α = 5% and β = 0.1, the sample size was 50 people in each intervention and control group, which increased to 60 people. For a sampling of four health centres in Delijan, two centres were randomly assigned (by drawing) to the intervention group and two centres to the control group. According to the recorded information in each centre, a list of people aged 30 years old–50 years old was extracted and each person was assigned a number. Later, using a table of random numbers, 60 people were selected for each group (30 people in each centre) and were invited by the health centre. In case the person did not go to the health centre, we went to their houses (Figure 1).

### Instruments

The data collection tool in this study was a valid and reliable researcher-made questionnaire. Data were demographic information questionnaires, stages of change, self-efficacy, processes of change, perceived benefits and barriers, and the International Physical Activity Questionnaire (IPAQ). The validity and reliability of IPAQ had been confirmed in a previous study in Iran (15). The reliability and validity of the other questionnaires have been confirmed by Marcus et al. (16) as well. Furthermore, these questionnaires were translated into Persian in the study of Roozbahani et al. (17) in Iran by translation-re-translation method, and their validity and reliability were confirmed. The reliability of the questionnaires in the study was evaluated by the test re-test method (IPAQ and Stages of Change) or Cronbach’s alpha coefficient (self-efficacy questionnaire, processes of change, and perceived benefits and barriers) according to their nature.

### International Physical Activity Questionnaire (18)

IPAQ was used to obtain information about the types of PA that people engage in as part of their daily lives. The IPAQ comprises four different detailed PA levels (work-related activity, leisure-time activity, transport-related activity, and domestic and garden activities) that require respondents to recall their PA over the past 7 days. In the present study, based on appropriate IPAQ items, individuals were classified as meeting PA recommendations (30 min of moderate recreational, walking or gardening activity 5 days per week or 20 min of vigorous recreational or gardening activity 3 days per week), being insufficiently active (not meeting recommendations but not completely inactive) or inactive (no moderate or vigorous recreational or gardening activities). The reliability and validity of IPAQ are evaluated in Iran (19). The reliability of this tool in the study was determined by test re-testing (*r* = 0.90).

### Stage of Exercise Behaviour Change Questionnaire

The stages of change were evaluated using the Stage of Exercise Behaviour Change Questionnaire (SECQS) of Marcus et al. (16). The participants were asked to show which of the following best described their present level of exercise behaviour (e.g. walking, swimming, cycling and playing ball sports for 30 min or more daily, 5 days a week): ‘I have been active for more than 6 months (maintenance), ‘I have been active for less than 6 months’ (action), ‘I am not regularly active, but I engage in activities occasionally and plan to start regularly the next month’ (preparation), ‘I am not active but am contemplation of starting in the next 6 months’ (contemplation) and ‘I am not active and not the contemplation of starting in the next 6 months’ (pre-contemplation). The reliability of this tool was determined by test re-testing (*r* = 0.92).

### Exercise Self-Efficacy Scale

The Exercise Self-Efficacy Scale (ESES), which was developed by Bandura (23), was used in the study. The scale has 18 items scored from 0 to 100. According to the study, individuals with a score of 0 were not confident in their skills of doing the exercise at all and those with 100 were most highly confident. Those moderately certain were the individuals with a score of 50. Items should be phrased in terms of ‘can do’ rather than ‘will do’ and should be written at the reading level of the participants. The phrases ‘I will do’ were replaced by ‘I can do’ in all items. In the current study, Cronbach’s alpha coefficient was 0.97.

### Processes of Change Questionnaire

The processes of change were measured using the Processes of Exercise Adoption (PEA) instrument. This questionnaire has 40 items (four items for each of the 10 processes of change) and uses a 5-point numeric response scale (1 = never; 5 = repeatedly). The score for each process of change has a range of 4 (low) to 20 (high). The participants were asked to recall the past month and rate the occurrence frequency of each item concerning the PA behaviour. Alpha coefficients for the study were good for the 40 items (α = 0.85).

### Decisional Balance Scale for Exercise

The exercise behaviour decisional balance questionnaire was used to evaluate the participants’ decisional balance. The scale has two sub-scales that show the positive (pros: 10 items) and the negative (cons: 6 items) aspects of PA behaviour. Participants were asked to show how significant each statement was regarding their decision whether or not to participate in PA behaviour. The scale is ranked from 1 = not at all important to 5 = extremely important. Cronbach’s coefficient alpha was used to evaluate internal consistency in the study (α = 0.94 for PA pros and α = 0.89 for PA cons).

### Designing Interactive Multimedia

Given the following reasons, it was decided to design a self-study package as multimedia software and present it to the intervention group. The target population was the individuals aged 30 years old–50 years old who could not quickly attend in-person training classes, either in groups or individually, given their busy lifestyles. Despite the training programme as virtual content, the individuals could use the training programme whenever they wanted and could. Each in-person training session is usually presented to the individual once. If the audience missed a part of the session because of absentmindedness or absence, it would be difficult for them to present the same content. In the multimedia part, the individuals could review the whole or part of the training session as many times as they wished. The probability of accepting that education will increase if the educational method is in line with the desire of the learners as much as possible. Although it is necessary to pay attention to the effect of the teaching method and its appropriateness to the educational objectives, one can state that the selected teaching method will have the best effect on the learners’ education if it is consistent with the desire of the participants. Given the reasons stated, the research group decided to provide the training package as a self-taught multimedia software so that the participants could have a complete training programme at home and meet their training needs with this package.

The name of this educational multimedia was selected as ‘Active Life’ and it was designed in six training sessions of approximately 15 min (one session per week) along with an introduction. Based on TTM, pre-contemplation and contemplation stages about PA behaviour, lesson plans were first written for each training session to design it. In writing the lesson plan, the principles of TTM were taken into account. For instance, in the first week, cognitive change processes were used and in the last week, behavioural change processes were used. Then the general goals, specific goals and behavioural goals of each session were written, and the strategies and educational activities of each session were specified. Besides, the principles of educational multimedia design were taken into account in designing multimedia. For instance, in the preparation of the educational programme, written text, simultaneous speech with text, related images, appropriate music and videos were used based on the principle of redundancy.

In each training session, activities (emotional, mental or physical active behaviour) or assignments were considered for the audience after presenting the training programme. The improvement in PA in these six sessions was gradual so that participants could eventually reach at least 150 min of moderate-intensity PA per week (30 min of activity per day and at least 5 days per week). The structures of TTM and the content of each session are given in Table 1.

### Intervention

The individuals in the intervention group were given multimedia training software designed based on TTM and a phone number to call in case they had any questions. The landline and mobile phone numbers of the participants were registered for further follow-up. The researchers provided no training during the intervention for the control group. Nonetheless, educational multimedia software was given to them to maintain medical ethics after the intervention and evaluations. The subjects were evaluated in two periods before the intervention and 6 months after it to evaluate the effectiveness of the training programme. In these two evaluations, the variables of change stages, change processes, self-efficacy and the perceived benefits and barriers of PA were examined using the questionnaires.

### Statistical Analysis

Statistical analysis was performed using IBM SPSS^®^ version 22 software. To measure the effects of each construct, a mixed factorial analysis of variance (ANOVA) at the 2-time points, was used. Time as the within-subject factor and group (intervention versus control) as the between-subject factor, as well as the interaction or main effect (time and group), were reported. Fisher’s exact test was used to evaluate the stages of change to compare the intervention group with the control group.

## Results

In the study, 120 people aged 30 years old–50 years old with a mean age of 38.20 (SD = 7.11) years old participated. The demographic characteristics of the intervention and control groups were the same at the beginning of the study (Table 2).

As one of the inclusion criteria was to be in the pre-contemplation or contemplation stage, at the beginning of the study, people in both groups were in one of these two stages, and the Fisher’s exact test did not show any differences between the two groups regarding this. Six months after the intervention, most members of the intervention group (61.7%) had progressed to the action and maintenance stages (*P* < 0.001). In the control group, most people (53.3%) were still in the pre-contemplation stage (Table 3).

Significant findings related to the effects on groups, times and interaction (group and time) from a mixed factorial ANOVA are reported for PA and TTM constructs, as seen in Table 4. Six months after the intervention, the mean weekly minutes of moderate-intensity PA and the scores of the transtheoretical model constructs of the intervention group were significantly higher than the control group.

## Discussion

The study examined the effectiveness of an intervention based on TTM designed to promote PA and the reported results showed the effectiveness of multimedia designed based on TTM on the promotion of PA. Significant changes in the constructs of TTM and the minutes of PA were observed in the participants who used this educational multimedia regularly for almost 100 min. The effective indices used in the study can be used to optimise the future of educational multimedia according to the needs and requirements of various groups.

In the present study, more progress was seen in the stages of changing the behaviour of PA in the intervention group compared to in the control group, so that this increase was higher in the intervention group (61.7%) compared to the control group (303%) progressed to the operation and maintenance stages 6 month after the intervention.

In a study by Mauriello et al. (20) to evaluate the effectiveness of using appropriate educational multimedia to promote PA, an increase in fruit and vegetable consumption, and limiting television viewing in 1,800 adolescents were implemented. Like this study, the progress of PA behaviour change in the intervention group was reported. In a study in Iran, a significant increase was observed in the levels of PA and stages of change in the intervention group after the intervention (21).

Regarding the moderate PA, there was a significant increase in weekly PA minutes in both intervention and control groups. However, this difference was greater in the intervention group compared to the control group. Using a theory-based educational multimedia tailored to the needs of the audience, as well as the use of TTM constructs, increased the effect on behaviour. An increase in minutes of PA was reported in the intervention group compared to the control group in the study by Leanne et al. (20). Furthermore, in a study conducted in Iran for evaluating theory-based educational multimedia on PA behaviour in Tehran women, a significant increase in moderate PA was observed 6 months after training (22).

Concerning the effect of educational intervention on TTM constructs, the individuals in the intervention group had a significant increase in self-efficacy of PA behaviour. In contrast, self-efficacy did not change in the control group. Bandura (23) states that as one of the most powerful tools for increasing self-efficacy is mastering behaviour, changes in self-efficacy may occur following the successful and active participation of individuals in PA. Moreover, self-efficacy may have a role in changing behaviour. In this study, the increase in self-efficacy of participants maybe because of using strategies used in educational multimedia, and besides increasing PA and success in achieving their goals, their self-efficacy has increased compared to before.

In a study entitled ‘The effect of health education programme based on TTM’ on the promotion of PA among the children of patients with hypertension and diabetes after the intervention, the mean score of self-efficacies in the stages of contemplation, acting and maintenance had a statistically significant difference between the two groups. The scores of the intervention group were higher than the control group (24). In a study by Hausenblas et al. (25), aimed at evaluating the effect of multimedia on women’s PA during pregnancy and postpartum, the self-efficacy of the intervention group increased compared to the control group. Moreover, the results of Romain et al. (26) in this regard are in line with those of our study. However, in a study that used a computer-based counselling system to enhance attitudes towards PA in patients with chronic disease, the increase in self-efficacy showed only minor changes (14, 27).

Furthermore, the results indicated that the subjects in the intervention group used more change processes in the 6 months after the intervention than the control group. In the study of Moodi et al. (27), done to determine the effect of training programme based on TTM, to increase the use of PA behaviour change processes in employees, a significant increase was observed in the mean score of the change process in the intervention group. The results of Jalilian et al. (28) to examine the effectiveness of the intervention programme based on TTM (as pamphlets, booklets and educational videos) in promoting PA in employees, confirms our study, so that the use of change processes had increased compared to pre-educational intervention after the educational intervention. According to a study entitled ‘Examining the effect of educational programme based on TTM on increasing the use of PA behaviour change processes among Birjand University employees immediately after the intervention,’ a significant increase was observed in the mean score of change processes in the intervention group compared to the control group. In addition, the mean score of 3 months after the intervention in the change processes showed a significant increase in the intervention group compared to the control group (27). The results of Liu et al. (29) are consistent with those of the present study. These results showed the positive effect of training on increasing the use of change processes.

Based on the studies conducted, the perceived benefits of the behaviour increase and the perceived barriers to performing that behaviour decrease when an individual moves from the pre-action stages to the action and maintenance stages of the behaviour (27). The results of the present study on the perceived benefits and barriers to PA approves of this. Thus, after the educational intervention, with the improvement of the change phase, the perceived benefits in the intervention group increased significantly, and the perceived barriers decreased, but. Still, in the control group, there were no significant changes in the perceived benefits and barriers. This has led to an increase in the number of people in the practice phase and subsequently decreased the number of people in the pre-contemplation phase. Reducing barriers in the intervention group can be interpreted as saying that with the problem-solving process strategy used in educational multimedia, participants were able to solve barriers to PA and thus increase their PA. Added and progressed in the stages of change. In addition, the findings of the present study are consistent with the results of similar studies (29–30).

It seems that the conditions of using location and time, issues that increase users’ acceptance of multimedia are important. Moreover, multimedia may be more useful than print-based media for people with limited literacy skills. The nature of educational multimedia enables its expansion and dissemination, which in turn leads to an increase in the number of active people in society.

The study had some limitations, the first of which is the possibility that results suffered from recall bias because of self-report. Furthermore, the study follow-up was 6 months. Longer follow-up may specify the effect of educational multimedia on the stability of better PA behaviour.

## Conclusion

Intervention by multimedia designed based on TTM was able to increase PA, progress in the stages of change. The main advantage of this multimedia is it is being design based on TTM. This process led to the training programme to be designed based on the real needs of the study population and thus increase the effectiveness of the training programme. Using TTM constructs in designing educational multimedia could provide effective training in relation to the promotion and sustainability of PA behaviour. According to the results of the study, it seems that the implementation of educational intervention using multimedia designed based on TTM could be used as one of the effective strategies to promote PA to prevent hypertension among the adults.

## Figures and Tables

**Table 1:** The structures of TTM and educational content in each multimedia session used in the intervention study

Main menu	TTM constructs	Educational content
Introduction	–	- the reason for preparing the programme - the familiarity of the learners with using multimedia
First week	- processes of change (consciousness raising) - decision balance (benefits of PA)	- defining PA - international guidelines for PA - the significance and benefits of PA
Second week	- processes of change (environmental re-evaluation, dramatic relief, and self-re-evaluation)	- the change in the behaviour of PA on the living environment of the individual and others - helping the learners to show the feelings and emotions related to the risk of inactivity - helping the learners to imagine themselves when they are inactive or, conversely, when they are active
Third week	- processes of change (self-liberation) - self-efficacy	- the types of PA in terms of intensity and conditions for doing it - designing long-term and short-term goals and commitment to reaching the goals - strategies to promote PA self-efficacy (a gradual increase of PA, use of observational learning and encouragement)
Fourth week	- decision balance (barriers to PA) - processes of change (social liberation)	- familiarity with the obstacles to PA and the problem-solving process - social opportunities are created to promote people’s PA
Fifth week	- processes of change (reinforcement management and helping relationships)	- management of reinforcement and self-reward - helping relationships and social support - attracting social support by learners
Sixth week	- processes of change (counter conditioning and stimulus control)	- learning new healthy behaviours instead of previous unhealthy ones - removing stimulants leading to unhealthy habits and quick addition of a healthy option

**Table 2:** Comparison of the intervention and control groups, concerning the demographic variables

Variables		Intervention		Control		*P*-value *t*-test

		mean	SD	mean	SD	
Age (years old)		37.31	7.17	39.58	8.16	0.174
BP (systolic)		114.91	13.96	113.54	14.89	0.777
BP (diastolic)		72.33	11.40	71.50	15.29	0.742

		**Number**	**%**	**Number**	**%**	***P*** **-value**

Education	Elementary school	9	15	12	20	0.710
	High school	32	53.3	32	53.3	
	Academic	19	31.7	16	26.7	
Sex	Male	32	53.3	31	51.7	0.855
	Female	28	46.7	29	48.3	
Marital status	Single	13	21.7	10	16.7	0.472
	Married	47	78.3	50	83.3	
Job	Full time	32	53.3	34	56.7	0.652
	Part time	15	25	11	18.3	
	Not in paid employment	13	21.7	15	25	

**Table 3:** Frequency of individuals in the stages of change before and 6 months after the intervention

Groups		Before intervention		6 months after intervention	

		Number	%	Number	%
Intervention	Pre-contemplation	32	53.3	0	0.0
	Contemplation	28	46.6	5	8.3
	Preparation	0	0.0	18	30.0
	Action	0	0.0	23	38.4
	Maintenance	0	0.0	14	23.3
Control	Pre-contemplation	33	55.0	32	53.3
	Contemplation	27	45.0	25	41.7
	Preparation	0	0.0	1	1.7
	Action	0	0.0	2	3.3
	Maintenance	0	0.0	0	0.0
	*P*-value	0.254		0.001	

**Table 4:** Mixed factorial ANOVA and *F*-statistics for physical activity and the constructed TTM

Variables	Groups	Before intervention		6 months after intervention		Test effect	*F*	*P*-value

		Mean	SD	Mean	SD			
						Time	68.84	0.001
Physical activity (minutes/week)	Intervention	36.02	31.22	146.16	90.43	Group	25.40	0.001
	Control	33.41	28.33	54.41	44.02	Time and Group	30.26	0.001
						Time	108.58	0.001
Processes of change	Intervention	103.1	31.40	141.7	13.31	Group	11.71	0.001
	Control	106.95	29.75	106.61	29.48	Time and Group	112.40	0.001
						Time	306.55	0.001
Self-efficacy	Intervention	15.73	13.80	56.42	13.24	Group	45.03	0.001
	Control	15.33	14.42	17.47	13.43	Time and Group	248.42	0.001
						Time	186.70	0.001
Cons	Intervention	16.61	4.57	9.78	3.02	Group	8.55	0.001
	Control	15.66	5.28	15.46	5.34	Time and Group	166.07	0.001
						Time	114.13	0.001
Pros	Intervention	32.60	8.37	42.80	6.66	Group	16.92	0.001
	Control	31.55	9.02	32.20	8.69	Time and Group	88.42	0.001
